# Multifunctional Bioplastics Inspired by Wood Composition: Effect of Hydrolyzed Lignin Addition to Xylan–Cellulose Matrices

**DOI:** 10.1021/acs.biomac.9b01569

**Published:** 2020-01-15

**Authors:** Giacomo Tedeschi, Susana Guzman-Puyol, Luca Ceseracciu, Uttam C. Paul, Pasquale Picone, Marta Di Carlo, Athanassia Athanassiou, José A. Heredia-Guerrero

**Affiliations:** † Smart Materials, Istituto Italiano di Tecnologia, Via Morego 30, Genova 16163, Italy; ‡ DIBRIS, Università di Genova, Via Opera Pia 13, Genova 16145, Italy; § Instituto de Hortofruticultura Subtropical y Mediterránea La Mayora, Universidad de Málaga - Consejo Superior de Investigaciones Científicas, Departamento de Mejora Genética y Biotecnología, Estación Experimental La Mayora, Algarrobo-Costa E-29750, Málaga, Spain; ∥ Materials Characterization Facility, Istituto Italiano di Tecnologia, Via Morego 30, Genova 16163, Italy; ⊥ Istituto per la Ricerca e l’Innovazione Biomedica (IRIB), Consiglio Nazionale delle Ricerche (CNR), Via Ugo La Malfa 153, Palermo 90146, Italy

## Abstract

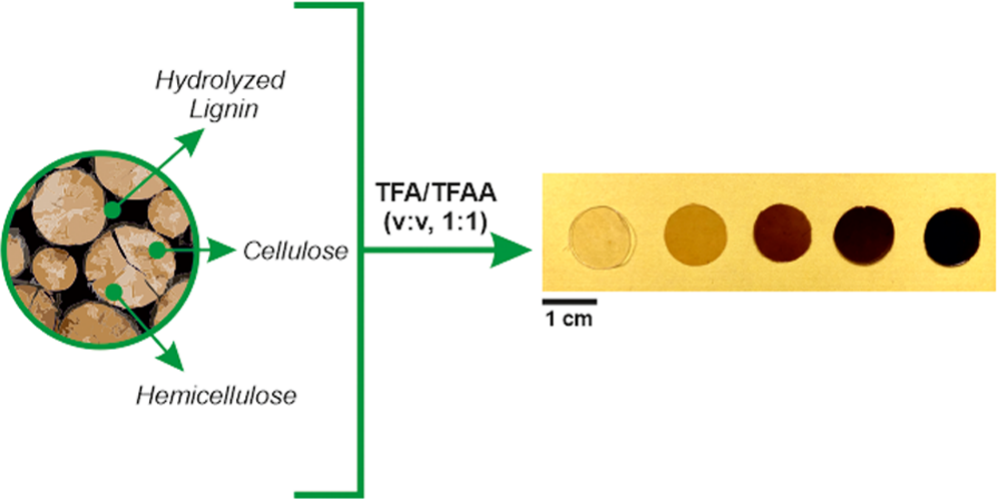

Multifunctional bioplastics have been prepared by amorphous reassembly of cellulose, hemicelluloses (xylan), and hydrolyzed lignin. For this, the biopolymers were dissolved in a trifluoroacetic acid–trifluoroacetic anhydride mixture and blended in different percentages, simulating those found in natural woods. Free-standing and flexible films were obtained after the complete evaporation of the solvents. By varying xylan and hydrolyzed lignin contents, the physical properties were easily tuned. In particular, higher proportions of hydrolyzed lignin improved hydrodynamics, oxygen barrier, grease resistance, antioxidant, and antibacterial properties, whereas a higher xylan content was related to more ductile mechanical behavior, comparable to synthetic and bio-based polymers commonly used for packaging applications. In addition, these bioplastics showed high biodegradation rates in seawater. Such new polymeric materials are presented as alternatives to common man-made petroleum-based plastics used for food packaging.

## 1 Introduction

Wood is a porous and fibrous structural tissue found in the stems and roots of trees and other woody plants. It is a natural composite material made of robust cellulose fibers that are embedded in a matrix of both hemicelluloses and lignin. The main components of wood are cellulose, hemicelluloses, lignin, and different extractives such as aliphatic/alicyclic compounds (terpenes, fatty acids, and alcohols), phenolic compounds, sugars, and alkaloids.^1^ Cellulose is the most abundant renewable polymer on earth, with a total annual biomass production of 1.5 × 10^12^ tons. It is composed of very long linear polymer chains of d-glucopyranosyl linked by β-1,4-glycosidic bonds. Cellulose is mainly used as a construction material, and for textiles, paper, and cardboard.^2^ Hemicelluloses consists of β-1,4-linked glycans with various substitutes. One of the most common subgroups of hemicelluloses are xylans whose chemical structure is made up of β-1,4-linked xylopyranosyl residues with side branches of α-arabinofuranose and α-glucuronic acids. Depending on the type of plant biomass, xylan can have different side groups and can be additionally chemically modified by acetylation. It contributes to the cross-linking of cellulose microfibrils and lignin through ferulic acid residues, in grasses, and through lignin-carbohydrate complexes in higher woody plants.^3−5^ Xylans are commercially used in different activities, including bread and livestock industries and as a natural food sweetener and second-generation fuel, to mention a few.^6^ Lignin is the second most abundant natural polymer after cellulose, with an annual production of ∼500 million tons.^7^ It is considered as the polymer matrix that provides rigidity, compressive strength, and protection against water from the external environment to the cell wall. Lignin is a highly branched and amorphous biopolymer with characteristic aromatic structures (i.e., monolignol monomers such as *p*-coumaryl, coniferyl, and sinapyl alcohols). It is used in different applications, for instance, as a filler in composite plastic materials, cosmetics, and fire retardants and as feedstock for biofuel production.^8^

Wood is employed in a broad range of different applications of human activities, from construction to fuels and tools. During its processing, wood industries generate enormous amounts of lignocellulosic biomass.^9^ To valorize this by-product in terms of a circular economy process, the combination with synthetic polymers to fabricate wood polymer composites (WPCs) has been proposed. The global WPC market was 4 billion USD in 2017, progressing at an estimated compound annual growth rate of 9.3% from 2018 to 2025.^10^ To produce WPCs, fine wood powder or woody biomass from agricultural residues are blended with polymers such as polyethylene, polyvinylchloride (PVC), or polypropylene (PP) within an extruder. The extruded material can then be pelletized for later processing by common industrial techniques, such as injection molding, extrusion, or compression molding. The wide acceptance of WPCs by the global market is due to their low-cost, lightweight, better properties than wood and plastic separately, easy recycling ability, adaptation to the existing plastic processing techniques, and the need for the plastic industry to move toward greener and more sustainable productions.^11^ This fast-growing WPC sector presents many new chances to exploit wood as a filler or reinforcement in polymer composites.^12^ One of them is the combination of wood filler with biodegradable and bio-based polymers such as poly(lactic acid) (PLA) and polyhydroxyalkanoates to replace common petroleum-based plastics in WPCs. The main reasons for this change can be found in the increasing concerns about the environmental impact of such petroleum-based polymers, the volume of plastic waste in landfills, and the more restrictive legislation that pushes toward a circular bioeconomy, particularly in Europe.^13^ The use of these bio-based polymers as matrices of WPCs potentially ensures the complete biodegradation of the composite. Hence, the applications of WPCs can be extended to relatively short-term (e.g., food packaging) and long-term uses (for example, pallets and furnishing), where recycling and ease of after-life management become an added value.^14^ So far, wood and its by-products from lignocellulosic biomass needed to be included in bio-matrices to develop materials useful for food packaging and related industries. Thus, the main objective of this work is to develop lignin-based materials, which can be used in such applications without the employment of other polymers as the matrix.

Trifluoroacetic acid (TFA) is a naturally occurring organic acid that can be biodegraded by microbial action.^15^ It is recyclable by distillation due to its high volatility and miscible with many organic solvents and water. It is one of the typical nonaqueous derivatizing solvents for cellulose.^16,17^ In the presence of TFA, cellulose is selectively trifluoroacetylated in the C6-hydroxyl groups and, subsequently, solved in the fluorinated acid.^18^ This derivative is readily hydrolyzed in water, water vapor, or the moisture in air, producing free-standing, amorphous, and transparent cellulose films. Recently, TFA has been exploited to fabricate bioplastic blends of cellulose with seaweeds, nylon, poly(vinyl alcohol), and nanocellulose.^19−22^ TFA has also been used as a solvent in the production of cellulose-based bioplastics from agro-wastes of edible vegetables and cereals. This process has been patented and proposed to be potentially scalable.^23^ Moreover, TFA has been combined with trifluoroacetic acid anhydride (TFAA), generating a reactive mixture that allows short times of solution and the acylation of cellulose and cellulose derivatives with carboxylic acids.^24,25^ Finally, it should be interesting to note that TFA and TFAA can also act as modifying agents in native wood tissues, causing the acid hydrolysis of pulp, or they can be employed as the catalyst for some chemical modification of wood (i.e., acetylation, esterification, etc.).^26^

In this work, we present a new methodology to fabricate transparent and multifunctional lignin-based bioplastics by reassembly of cellulose, hemicelluloses, and hydrolyzed lignin by simple solution in trifluoroacetic acid (TFA)–trifluoroacetic anhydride (TFAA) as a cosolvent system and subsequent evaporation at room conditions. In particular, different contents of these biopolymers, simulating the chemical composition of natural woods, were tested. Differences in optical, chemical, mechanical, thermal, hydrodynamic, and barrier properties among the samples are reported. Moreover, we show how the increasing lignin content modifies all of these features and provides antioxidant and antimicrobial properties to such lignin-based bioplastics.

## 2 Experimental Section

### 2.1 Materials

High-purity microcrystalline cellulose (crystallinity ∼79%, product number 435236, *M*_n_ ∼ 16 000 g/mol determined by SEC) from cotton linter pulp, hydrolyzed lignin (alkali, kraft, with low sulfonate content, product number 471003) from the Kraft pulping process, 2,2′-azinobis(3-ethylbenzothiazoline-6-sulfonic acid) diammonium salt (ABTS), potassium persulfate, 6-hydroxy-2,5,7,8-tetramethylchromane-2-carboxylic acid (Trolox), trifluoroacetic acid (TFA), and trifluoroacetic anhydride (TFAA) were purchased from Sigma-Aldrich. Xylan (from corn core, product number X0078, 75.0% minimum xylose content) was purchased from TCI Europe. All of the materials and the solvents were used without any further purification. *Escherichia coli* (ATCC 25922) was used for the antimicrobial test, and Lysogeny broth (LB) agar plates and LB liquid medium were purchased from USB Corporation (Cleveland, OH).

### 2.2 Preparation of Lignin-Based Bioplastic Films

The preparation of lignin-based bioplastics was carried out through the following procedure. First, separated solutions of cellulose, hydrolyzed lignin, and xylan were prepared by dissolving 1.5 g of these polymers in 100 mL of TFA/TFAA (2:1, v/v) mixture in 150 mL closed flasks and stirring at 80 °C for 45 min. Then, the starting solutions were blended together by mixing the corresponding volumes of each one to achieve the chemical compositions described in Table 1. After 5 min of magnetic stirring at 80 °C, the starting solutions became homogeneous. Then, 25 mL of the final solutions were drop-casted on glass Petri dishes (9 cm diameter) and kept under a chemical hood until the complete evaporation of the solvent (3 days). After this, free-standing films with an average thickness of ∼80 μm were obtained. The films were washed with water and cold methanol three times for each solvent and dried at room temperature for 24 h under vacuum to remove any residual solvent. The final cellulose content in the samples was 50 wt % of the total, while hydrolyzed lignin and xylan contents were varied accordingly from 0 to 50 wt %. For reasons of clarity and readability, samples were labeled as CLX-*x* (C: cellulose, L: hydrolyzed lignin, and X: xylan), where *x* is the percentage of hydrolyzed lignin, as explained in Table 1. Control samples of pure biopolymers were prepared by following the same procedure, but without blending. Cellulose (labeled as C) and xylan (X) formed free-standing films, while hydrolyzed lignin (L) did not form films, as shown in Figure S1.

**Table 1:** Labeling and Different Proportions of Cellulose, Hydrolyzed Lignin, and Xylan of the Samples Used in This Work

label	cellulose (wt %)	hydrolyzed lignin (wt %)	xylan (wt %)
C	100	0	0
X	0	0	100
L	0	100	0
CLX-0	50	0	50
CLX-12.5	50	12.5	37.5
CLX-25	50	25	25
CLX-37.5	50	37.5	12.5
CLX-50	50	50	0

### 2.3 Characterization

#### 2.3.1 Morphological Characterization

Scanning electron microscopy (SEM) images were acquired using a JEOL JSM-6490OLA, operating at 10 kV acceleration voltage. All of the samples were coated with a 10 nm thick film of gold. To analyze the top-view and the cross-sectional morphology of the samples, imaging operation was carried out with secondary electrons.

#### 2.3.2 Structural Characterization

X-ray diffraction (XRD) measurements were performed using a PANalytical Empyrean X-ray diffractometer equipped with a 1.8 kW Cu Kα ceramic X-ray tube, PIXcel3D 2 × 2 area detector, operating at 45 kV and 40 mA. The diffraction patterns were collected in parallel-beam geometry and a symmetric reflection mode using a zero-diffraction silicon substrate over an angular range: 2θ = 10–70°, with a step size of 0.03° and a scan speed of 0.1° s^–1^.

#### 2.3.3 Chemical Characterization

Infrared spectra were obtained with a single-reflection attenuated total reflection (ATR) accessory (MIRacle ATR, PIKE Technologies) coupled to a Fourier-transform infrared (FTIR) spectrometer (Equinox 70 FTIR, Bruker). All spectra were recorded in the range from 3800 to 800 cm^–1^ with a resolution of 4 cm^–1^, accumulating 128 scans. To assess the homogeneity of chemical composition, ATR-FTIR spectra were recorded three times in three different areas.

#### 2.3.4 Optical Characterization

Transparency was determined as the normalized transmittance according to the standard ASTM D1746 using a UV spectrophotometer Varian Cary 6000i.^27^ For this, bioplastics were cut into a rectangle piece and directly placed in the spectrophotometer test cell. An empty test cell was used as a reference. Five measurements were taken from different samples, and the results were averaged to obtain a mean value. Normalized transmittance, in percentage, was calculated as indicated below^28^
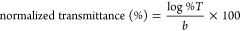
where %*T* is the transmittance at 600 nm, and *b* is the thickness of the sample (mm).

#### 2.3.5. Mechanical and Thermal Characterization

The mechanical properties of samples were characterized by uniaxial tensile tests on a dual column Instron 3365 universal testing machine equipped with a 500 N load cell. The tensile measurements were conducted according to ASTM D 882 Standard Test Methods for Tensile Properties of Thin Plastic Sheeting. For this, dog-bone shaped samples (25 mm length, 4 mm width) were stretched at a rate of 2 mm/min. Stress–strain curves were recorded at 25 °C and 44% relative humidity (RH). A minimum of seven measurements was carried out for each sample, and the results were averaged to obtain a mean value. The values of Young’s modulus, toughness (taken as the area below the curve, i.e., the fracture energy), stress, and elongation at break were calculated from the stress–strain curves.

The thermal degradation behavior of lignin-based bioplastic samples was investigated through thermogravimetric analysis (TGA) using a Q500 analyzer from TA Instruments. The measurements were carried out under an inert N_2_ atmosphere on 3 mg samples in an aluminum pan at a heating rate of 10 °C/min, from 30 to 600 °C. The weight loss (TG curve) and its first derivative (DTG curve) were recorded simultaneously as a function of time and temperature.

#### 2.3.6 Wettability and Hydrodynamical Characterization

To characterize the surface wettability of the samples, static water contact angles (W-CAs) were measured by the sessile drop method at room temperature at five different locations on each surface using a contact angle goniometer (DataPhysics OCAH 200). Then, 5 μL droplets of Milli-Q water were deposited on the surfaces and side-view images of the drops were captured. W-CAs were automatically calculated by fitting the captured drop shape and after 2 min from the drop deposition on the surface to consider values at equilibrium.

Water uptake measurements were also carried out. Dry samples were weighed on a sensitive electronic balance (0.0001 g accuracy) and placed in different humidity chambers. The different humidity conditions were changed before each weighing and set, respectively, at 0 and 100%. After remaining in the humidity chambers for one day, each sample was weighed and the amount of adsorbed water was calculated based on the initial dry weight as the difference.

#### 2.3.7 Barrier Properties

Water vapor permeability (WVP) was determined at 25 °C and under 100% relative humidity gradient (ΔRH %) according to the ASTM E96 standard method. In this test, permeation chambers with a 7 mm inside diameter and a 10 mm inner depth were used and filled with 400 μL of deionized water (which generates 100% RH inside the permeation cell).^20^ The samples were cut into circles and mounted on the top of the permeation chambers. The permeation chambers were placed in a desiccator with anhydrous silica gel, which was used as a desiccant agent to maintain 0% RH. The water transferred through the film was determined from the weight change of the permeation chamber every hour during a period of 8 h using an electronic balance (0.0001 g accuracy) to record mass loss over time. The mass losses of the permeation chambers were plotted as a function of time. The slope of each line was calculated by linear regression. Then, the water vapor transmission rate (WVTR) was determined as indicated below^29^
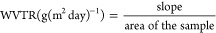
WVTR and water vapor permeability (WVP) measurements were replicated three times for each film. The WVP of the sample was then calculated as follows
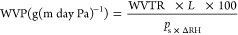
where *L* (m) is the thickness of the sample, measured with a micrometer with 0.001 mm accuracy, ΔRH (%) is the percentage relative humidity gradient, and *p*_s_ (Pa) is the saturation water vapor pressure at 25 °C.^30^

The oxygen permeation tests were performed using an Oxysense 5250i device (Oxysense) equipped with a film permeation chamber. This machine was operated according to an ASTM method F3136-15 (ASTM 1989).^31^ The test was performed at room conditions (23 °C, 50% RH). The permeation chamber consisted of a cylinder divided into two parts (sensing well and driving well). The sensing well was instrumented with a fluorescence sensor called Oxydots, sensitive to the oxygen concentration. This chamber was purged with nitrogen, while the other one (driving well) was kept open to ambient air. The films were cut into rectangular pieces (6 × 6 cm^2^) and placed inside the chamber. The OxySense fiber-optic pen measures the oxygen reading from the Oxydots, at specific time intervals. The oxygen transmission rate (OTR) of the films was measured by monitoring the oxygen uptake with time. Oxysense OTR software used this oxygen evolution to determine the OTR of the samples. At least ten readings were taken for each sample with a minimum coefficient of determination (*R*^2^) value of 0.995.

Grease resistance tests were carried out according to the Tappi test method T 559 cm-12. This is a standard procedure for testing the degree of grease repellency of paper and paperboard. In this test, 12 different liquids composed of castor oil, toluene, heptane, and their mixture at specifically defined weight ratios were used. Depending on the specific proportions of the three reagents, each solution was identified with a number ranging from 1 to 12, called Kit numbers (1–12). Kit number 1 is the least aggressive oil, while Kit number 12 is the most aggressive oil. To assess the grease resistance, drops of various solutions are placed on the surface of the bioplastic samples from a height of 2.54 cm (1 inch) and gently removed after 15 s. By visual inspection of the trace left or not on the surface, the substrate can be classified as grease resistant or not. The highest numbered solution that remained on the surface of the sample without causing staining is reported as the Kit value for the sample. The higher Kit value indicates the higher oil resistance of the sample. Ten measurements were performed for each sample, and the results were averaged to obtain a mean value.

#### 2.3.8 Antioxidant and Antimicrobial Properties

The antioxidant capacity was measured following the ABTS free radical cation scavenging assay. The ABTS radical cation (ABTS^•+^) was generated by the reaction between 7 mM ABTS solution with 2.45 mM potassium persulfate solution in the dark at room temperature for 12–16 h.^32^ The ABTS^•+^ solution was diluted with water to obtain an absorbance of 0.80 au at 734 nm. After that, 5 × 5 mm^2^ films were added to 3 mL of diluted ABTS^•+^ solution. The decrease in absorbance was determined at 734 nm with a Cary JEOL spectrophotometer at different times. Trolox, a water-soluble analogue of vitamin E, was used to prepare the calibration curve. The measurements were performed in triplicate, and the results were averaged to obtain a mean value. Radical scavenging activity was expressed as the inhibition percentage of free radical by the sample and calculated as follows
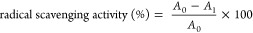
where *A*_0_ is the absorbance value of the control radical cation solution and *A*_1_ is the absorbance value of the sample at different times. Curves were normalized according to the hydrolyzed lignin present in the samples.

Antimicrobial activity of CLX-0, CLX-12.5, CLX-25, CLX-37.5, and CLX-50 samples and of C, L, and X were tested against *E. coli*. The infiltration test was performed according to Picone et al.^33^ First, pieces of the films (1 cm diameter) were sterilized under a UV lamp at 254 nm for 30 min and placed on an LB agar plate. Then, 2 μL of a diluted (1:10^5^) *E. coli* overnight culture solution was dropped on the center of the biomaterial surface, and 2 μL of the same bacterial solution was dropped directly on an LB plate, which was used as a control. Then, all of the plates were incubated at 37 °C overnight. Pieces of agar of about 0.3 cm diameter from the area under the biomaterials and from the control were punched and incubated in the LB liquid medium. After 2 h, the bacterial growth was determined by spectrophotometric measurement (OD_600_).

#### 2.3.9 Biodegradation Tests

Biodegradability was evaluated on selected samples through a standard biochemical oxygen demand (BOD) test by measuring the oxygen amount consumed during biodegradation in water.^34^ For each sample, three measurements were collected and the results were averaged to obtain a mean value. Weighed samples (∼200 mg) were minced and immersed in 432 mL bottles containing seawater collected from the Genoa (Italy) area shoreline. Oxygen consumed during the biodegradation process was recorded at different time intervals using sealed OxyTop caps on each bottle, which can assess the oxygen levels. BOD from blank bottles filled with only seawater was also measured as controls.

#### 2.3.10 Statistical Analysis

All of the measurements were carried out at least in triplicate or in a higher number (depending on the particular characterization analysis) to have an average value with a standard deviation. For the antimicrobial tests, the significance of the differences in the mean values of multiple groups was evaluated using analysis of variance. Differences were considered significant when the *p*-value was ≤0.05.

## 3 Results and Discussion

### 3.1 Morphological and Chemical Characterization

**Figure 1:**
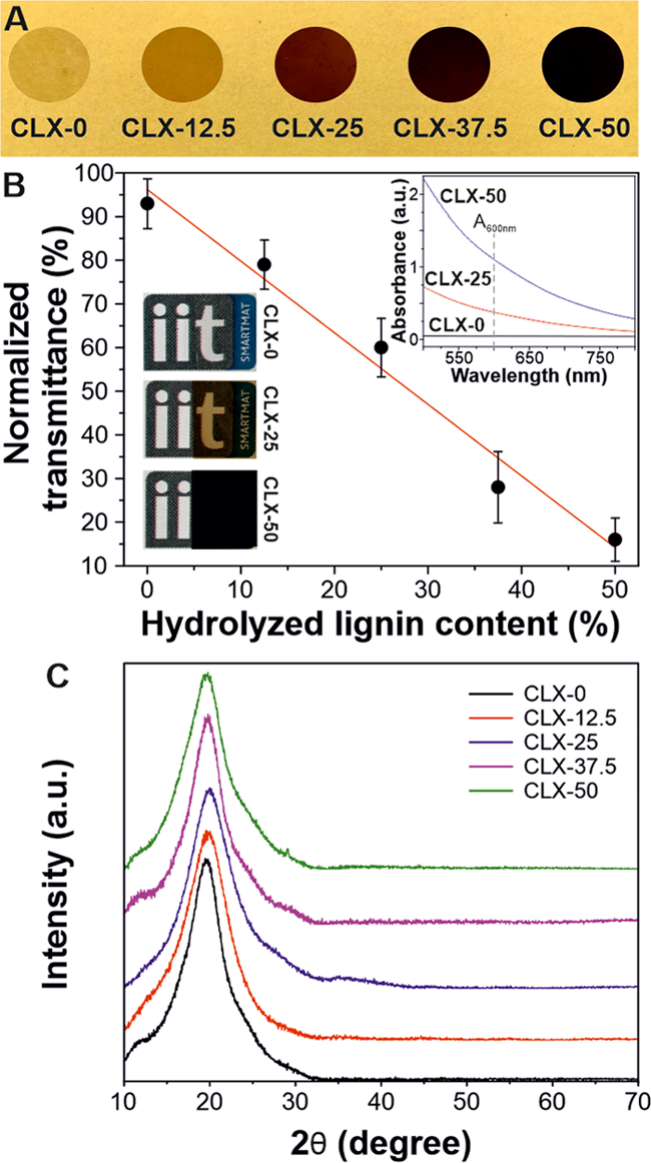
(A) Photographs of lignin-based bioplastic materials. (B) Normalized transmittance measurements of lignin-based samples. Top inset: UV–vis spectra of CLX-0, CLX-25, and CLX-50 samples with the same thickness in the range of the visible light spectrum. Bottom insets: macroscopic appearance of CLX-0, CLX-25, and CLX-50 samples (credits for the logo to Istituto Italiano di Tecnologia). (C) XRD patterns of lignin-based bioplastics.

Figure 1A shows a photograph with all of the lignin-based bioplastics (the final appearance of control samples is shown in Figure S1). CLX-0 was a colorless, highly transparent film, typical of cellulose and xylan amorphous dry films, Figure 1A and bottom inset of Figure 1B.^35^ As expected, the addition of hydrolyzed lignin induced a decrease of transparency and darker color in the samples, most probably because of the presence of visible radiation-absorbing aromatic repetitive units in its chemical structure (scattering phenomena have not been considered since the cross-section and surfaces showed very similar features, Figure S2). The color ranged from yellowish-brownish for CLX-12.5 to dark brown-black for CLX-50. Figure 1B presents the normalized transmittance values as a function of the hydrolyzed lignin content. Transmittance values linearly decrease with the hydrolyzed lignin content from 93% (high transparency) for CLX-0 to 16% for CLX-50 (high opacity). This increase in the opacity was also observed as a rise of the background in the UV–vis spectra of CLX films, inset of Figure 1B.

The structure of the lignin-based bioplastics was also characterized by XRD diffraction analysis, Figure 1C. The XRD patterns did not show significant differences between the different lignin-based bioplastics. All of the samples displayed a similar amorphous behavior with a broad halo centered at ∼19–20°.^19^ Nevertheless, the pure lignin control sample, L, showed characteristic diffraction peaks in the 2θ angle range between 20 and 40° and between 50 and 70°. These peaks are directly related to the acid hydrolysis of lignin.^36^ In fact, they correspond to some remaining inorganic salt crystals, i.e., sodium sulfate minerals, in the final hydrolyzed lignin, as shown in the peak assignment in Figure S3.

**Figure 2:**
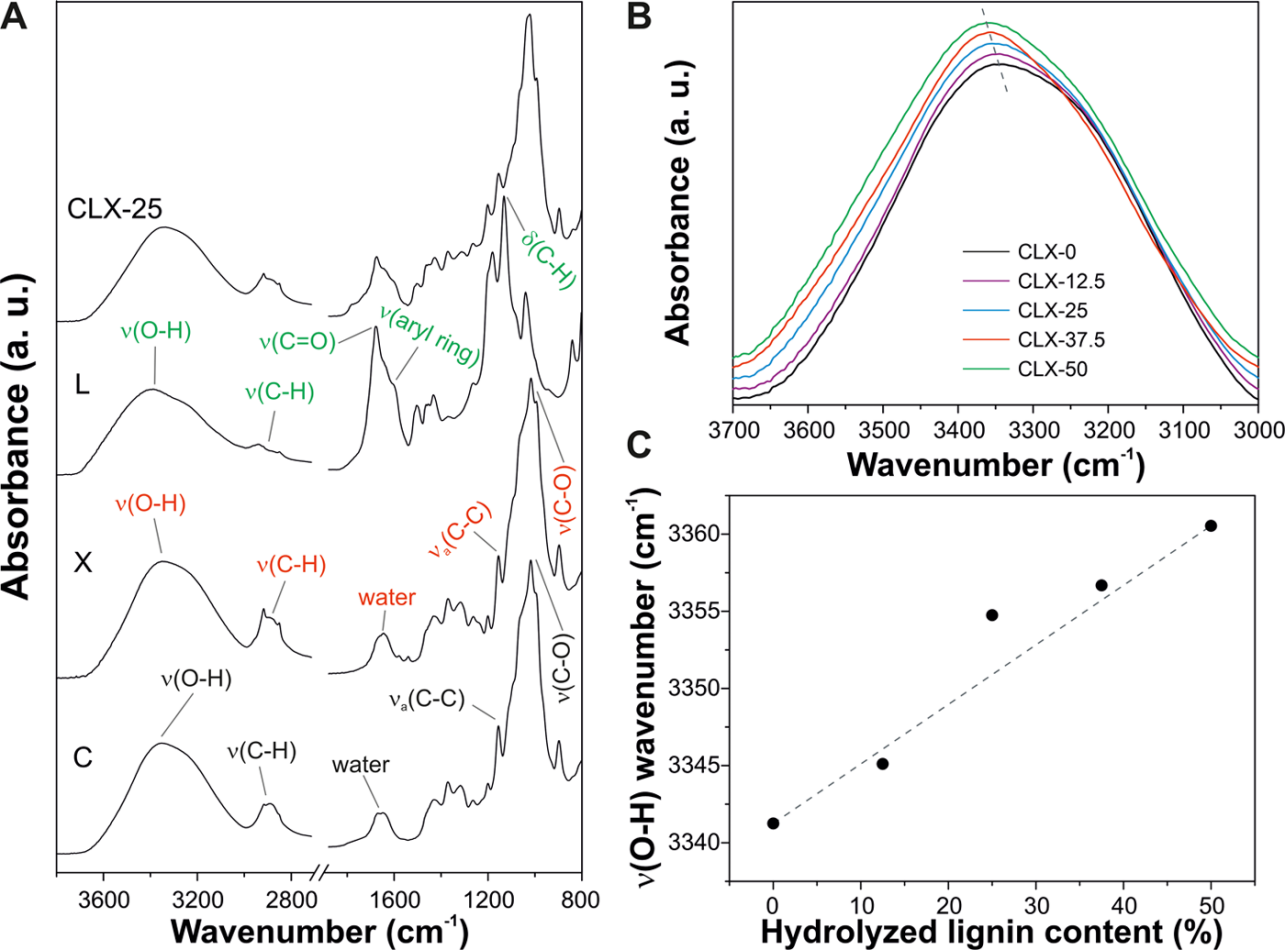
(A) ATR-FTIR spectra of C, X, L, and CLX-25 samples. (B) O–H stretching mode region of CLX samples. (C) Shift of the O–H stretching mode with the hydrolyzed lignin content.

The samples and the pure cellulose, hydrolyzed lignin, and xylan were chemically characterized by ATR-FTIR spectroscopy, Figure 2. Figure 2A shows the infrared spectra of C, L, X, and, as a representative example of the blends, CLX-25. Main vibrations of cellulose were assigned to the O–H stretching mode at 3349 cm^–1^, different C–H and CH_2_ stretching modes in the region between 2985 and 2810 cm^–1^, adsorbed water at 1655 cm^–1^, antisymmetric in-phase ring stretching at 1155 cm^–1^, and the C–O stretching mode at 1017 cm^–1^.^24^ Xylan presented a very similar spectral pattern: the O–H stretching mode at 3339 cm^–1^, different C–H and CH_2_ stretching modes in the region between 2990 and 2820 cm^–1^, adsorbed water at 1655 cm^–1^, antisymmetric in-phase ring stretching at 1157 cm^–1^, and the C–O stretching mode at 1018 cm^–1^.^37^^37^ On the other hand, the main bands of hydrolyzed lignin were: the O–H stretching mode at 3389 cm^–1^, different C–H and CH_2_ stretching modes in the region between 2990 and 2815 cm^–1^, a ring-conjugated C=O stretching mode at 1678 cm^–1^, a symmetric aryl ring stretching mode at 1601 cm^–1^, and aromatic C–H in-plane deformation at 1132 cm^–1^.^38^ CLX-25 showed infrared bands associated with the three components. Interestingly, a shift of the O–H stretching mode for the CLX samples was observed, Figure 2B. The wavenumber of ν(O–H) was increased from 3341 cm^–1^ for CLX-0 to 3361 cm^–1^ for CLX-50 (i.e., an increase of 20 cm^–1^), Figure 2C. Such a shift of this band to higher wavenumbers is indicative of weaker H-bonds.^39^ In this sense, the incorporation of hydrolyzed lignin into cellulose–xylan blends induces the rupture of hydrogen bonds between both polysaccharides. Most likely, this is caused by the different chemical natures of the hydrophilic polysaccharides and the hydrophobic hydrolyzed lignin.

### 3.2 Mechanical and Thermal Characterization

**Figure 3:**
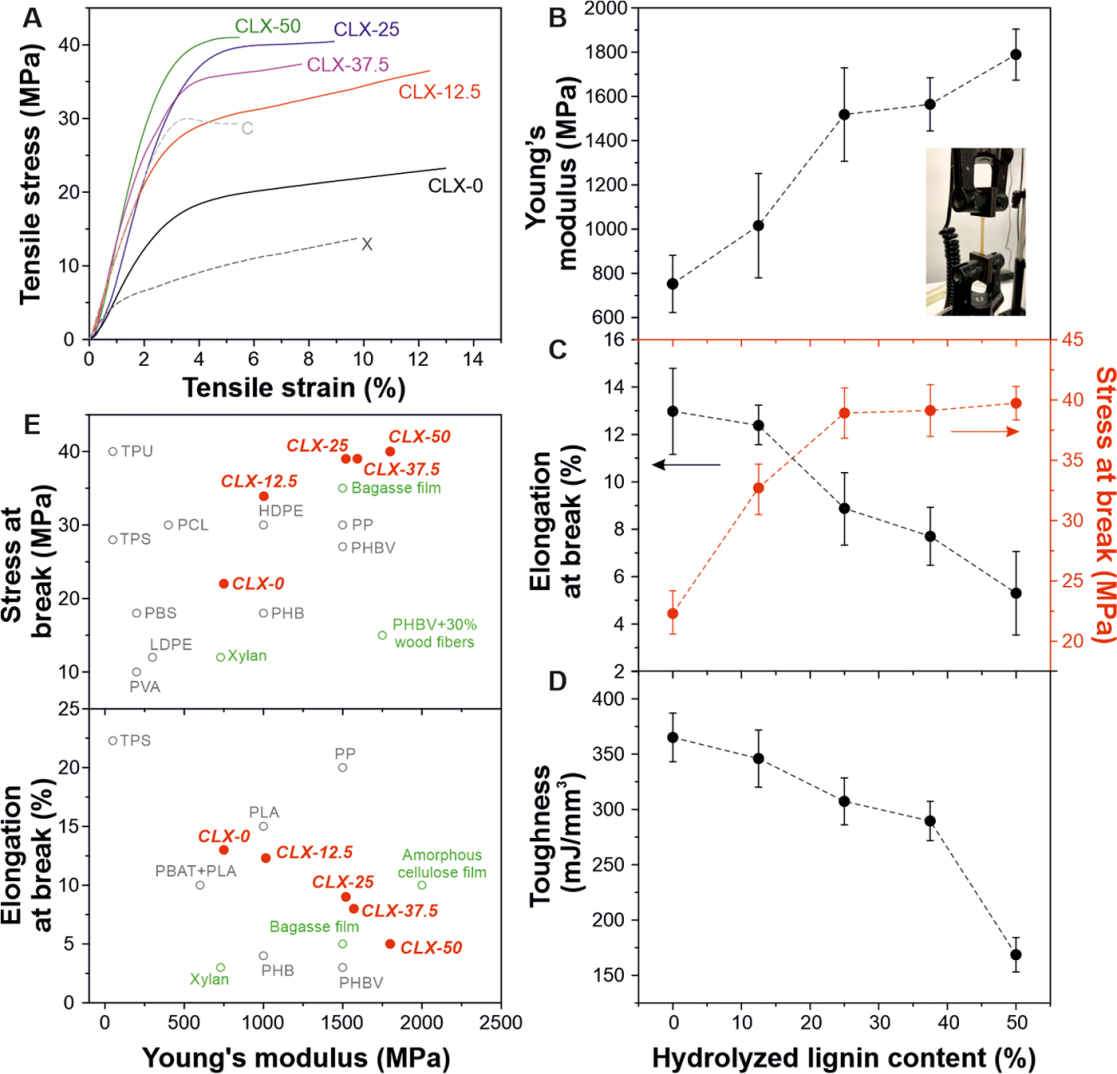
(A) Typical stress–strain curves of CLX-0, CLX-12.5, CLX-25, CLX-37.5, and CLX-50 films and of C and X control samples. (B, C, D) Young’s modulus, elongation, and stress at break, and toughness parameters, respectively, for the lignin-based bioplastics. The inset in B shows a photo of a lignin-based bioplastic during a tensile test. (E) Ashby plots of stress and elongation at break versus Young’s Modulus data for CLX-0, CLX-12.5, CLX-25, CLX-37.5, and CLX-50 samples in comparison to petroleum-based and bio-based plastics (gray) such as high-density polyethylene (HDPE), low-density polyethylene, polypropylene (PP), thermoplastic polyurethane, thermoplastic starch, polycaprolactone, poly(vinyl alcohol) (PVA), and polyhydroxybutyrate (PHB) and to lignin-based composite materials (green) (i.e., bagasse film, xylan film, amorphous cellulose film, and PHB with wood fibers).^11,21,43−45^

The mechanical properties of lignin-based bioplastics are presented in Figure 3. The typical stress–strain curves are displayed in Figure 3A, while the calculated mechanical parameters (i.e., Young’s modulus, elongation, and stress at break, and toughness) are reported in Figure 3B–D. As shown in Figure 2A, C and X control films presented different mechanical behaviors with a rigid behavior for C and a higher ductility for the X film. Nevertheless, the addition of hydrolyzed lignin modified the mechanical behavior from relatively good ductility for CLX-0 and CLX-12.5 characterized by lower Young’s moduli (∼753 and ∼1015 MPa, respectively) and stress at break values (∼22 and ∼32 MPa, respectively) and higher elongations at break (∼13 and ∼12.4%, respectively) to a rigid behavior for CLX-25, CLX-37.5, and CLX-50 samples with higher Young’s moduli (∼1518, ∼1563, and ∼1789 MPa, respectively) and stress at break (∼38, ∼39, and ∼39 MPa, respectively) and lower values of elongation at break (∼8.8, ∼7.7, and ∼5.3%, respectively). Similar to natural wood, this variation can be ascribed to the plasticizing effect of xylan and the stiffness and rigidity provided by the lignin as well as to the changes in the secondary H-bond network, as characterized by ATR-FTIR spectroscopy.^40,41^ Furthermore, the toughness of CLX films decreased with the hydrolyzed lignin content from ∼365 mJ/mm^3^ for the CLX-0 sample to ∼170 mJ/mm^3^ for the CLX-50 sample, as shown in Figure 3D. This could be explained by the dependence of toughness on the ductility of the materials. Therefore, in these lignin-based bioplastics, xylan is also responsible for the increase in the toughness values.^42^Figure 3E compares the values of stress and elongation at break versus the Young’s modulus data for CLX samples with other common polymers and petroleum-based plastics. The CLX lignin-based bioplastics with higher lignin contents (i.e., CLX-25, CLX-37.5, and CLX-50) presented the higher Young’s modulus and stress at break values than polymers like PP, high-density polyethylene (HDPE), polyhydroxybutyrate-*co*-valerate, and composite materials derived from wood (such as xylan or bagasse films and polyhydroxybutyrate (PHB) with wood fibers). On the other hand, regarding the Ashby plot of elongation at break versus the Young’s Modulus data, CLX samples covered the gap between polyhydroxybutyrates and PLA, PP, and amorphous cellulose. Therefore, these lignin-based bioplastics displayed competitive mechanical properties, complementary to commercial polymers and man-made plastics.

**Figure 4:**
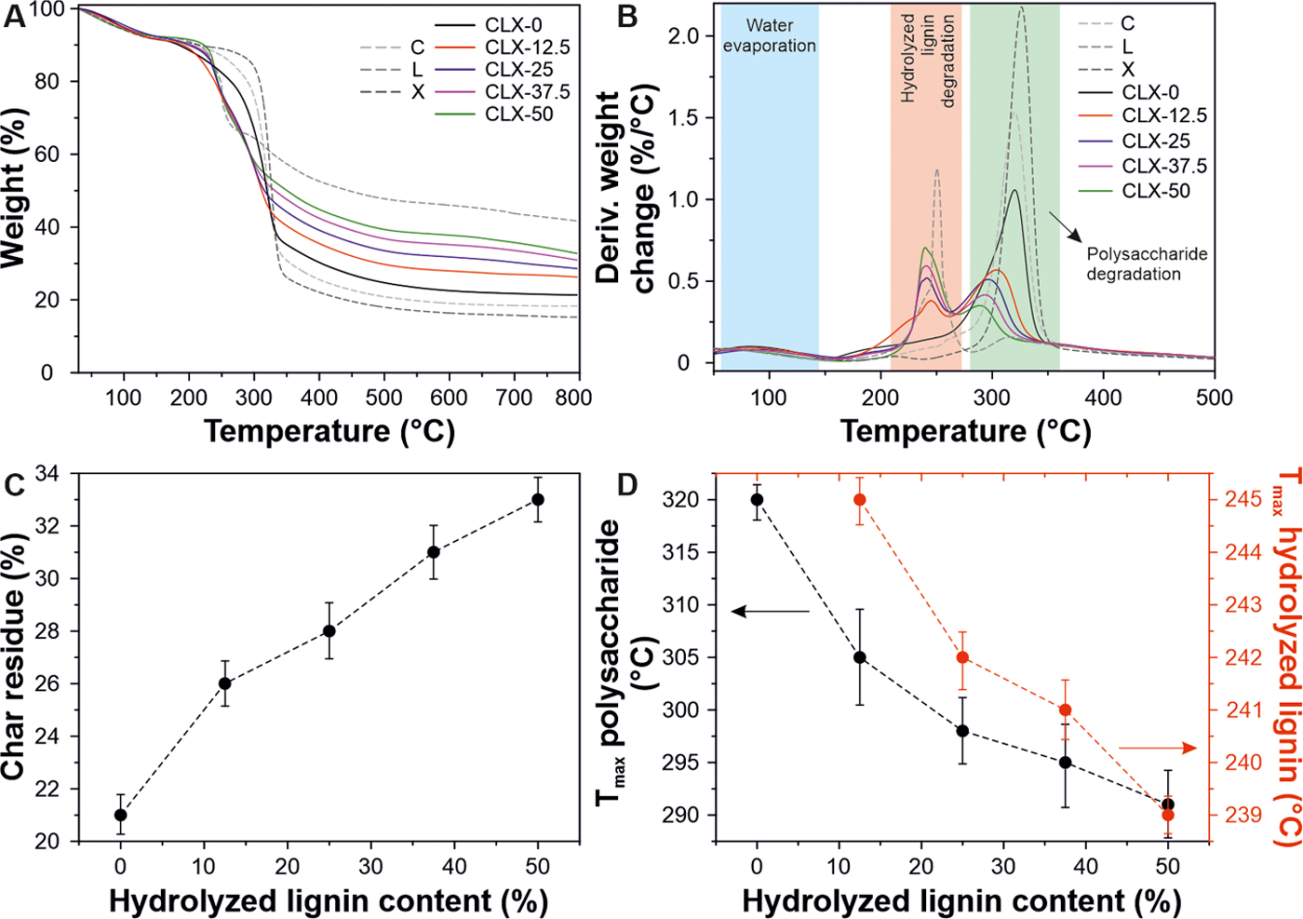
(A, B) TGA thermograms and derivative thermogravimetric curves of lignin-based samples (CLX-0, CLX-12.5, CLX-25, CLX-37.5, and CLX-50) and control samples (C, X, and L). (C) Char residue of the lignin-based samples after 800 °C as a function of hydrolyzed lignin content. (D) Shift of the temperature of the maximum mass loss rate related to lignin (red) and polysaccharide (black) degradations with respect to the hydrolyzed lignin content.

The thermal stability of the composite samples and controls (C, L, and X) was evaluated by TGA, Figure 4. All samples presented a first weight loss at ∼80 °C that can be related to the water evaporation and corresponded to ∼5% of the total weight. As shown, the L sample exhibited a single weight loss at ∼250 °C. Such a thermal event has been previously reported for the decomposition of hydrolyzed lignin.^46^ On the other hand, cellulose and xylan exhibited a single weight loss attributed to their thermal degradation at ∼326 and ∼320 °C, respectively.^47^ The lignin-based bioplastics showed two weight losses, the first one between 239 and 242 °C associated with the lignin decomposition and the second one between 289 and 320 °C related to the polysaccharide degradation. Char residue values of the lignin-based bioplastics after the thermal degradation process are presented in Figure 4C. As expected, the char content was higher as the hydrolyzed lignin percentage was increased. Finally, a small shift of *T*_max_ of polysaccharide and lignin fractions to lower temperatures with the hydrolyzed lignin content was observed, Figure 4D. As described above, the polysaccharide fraction needs a higher temperature to degrade with respect to the hydrolyzed lignin fraction. Consequently, the peak shift can be related to the less heat required for the cracking of lignin-based bioplastics with a higher amount of hydrolyzed lignin.^48^

### 3.3 Hydrodynamic and Barrier Properties

**Figure 5:**
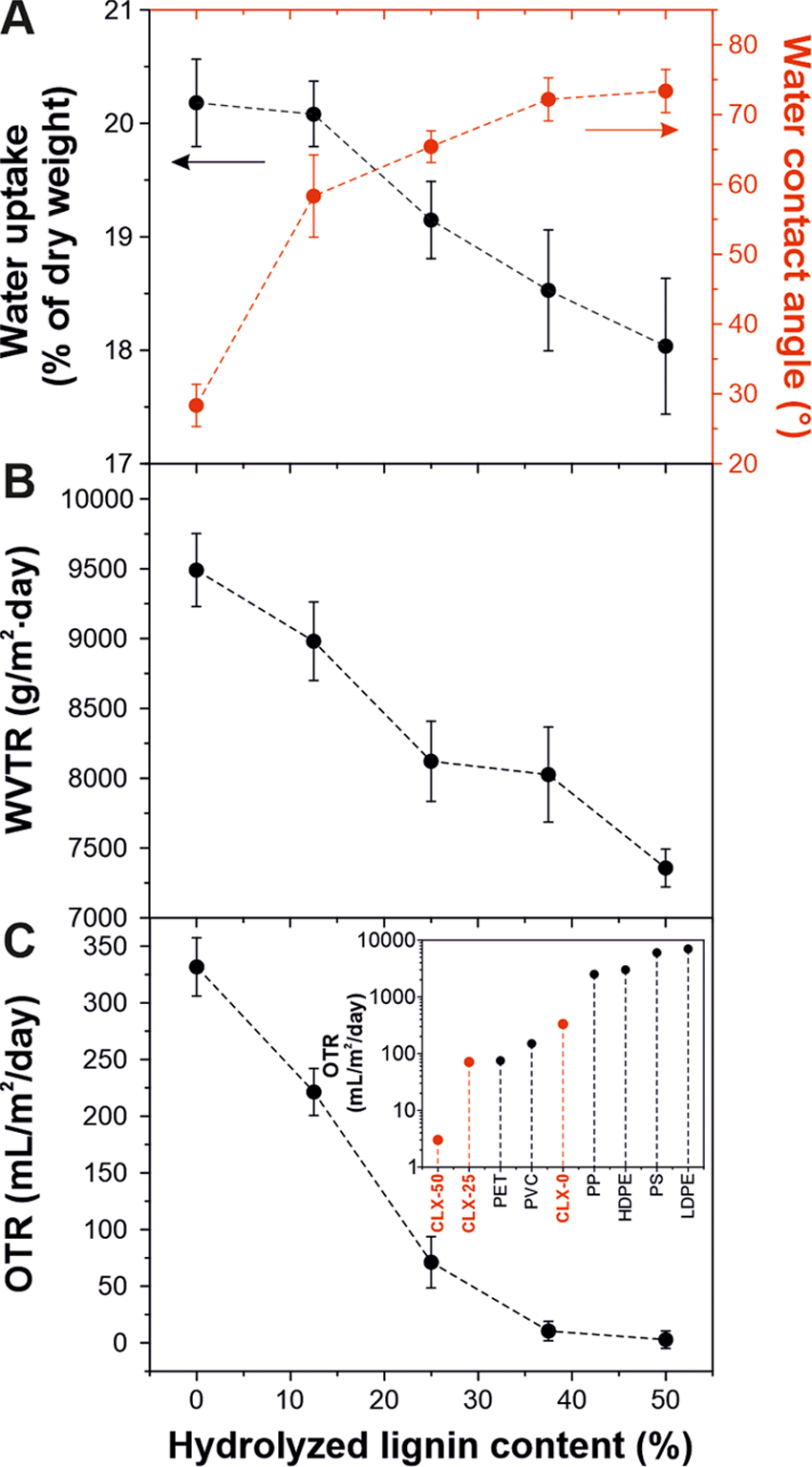
(A, B, C) water uptake and water contact angle (W-CA), water vapor transmission rate (WVTR), and oxygen transmission rate (OTR) values, respectively, for lignin-based bioplastics as a function of the hydrolyzed lignin content. Inset of (C): OTR values of CLX samples in comparison to common petroleum-based plastics for packaging.

The analysis of wettability, water uptake, water vapor, and oxygen barrier properties is shown in Figure 5. The values of static water contact angles are displayed in Figure 5A. Although the main fraction of the lignin-based bioplastics are polysaccharides, there is an increase of W-CA from 28° for CLX-0 to 73° for CLX-50 induced by the polyaromatic structure of hydrolyzed lignin.^49^ Likewise, the water uptake at 100% RH decreased linearly (∼11%, from CLX-0 to CLX-50) with the hydrolyzed lignin content, as displayed in Figure 5A. The partial substitution of hydrophilic polysaccharide -OH groups by aromatic structures of hydrolyzed lignin can explain this phenomenon. Regarding water vapor barrier properties, the water vapor transmission rate (WVTR) is reported in Figure 5B. These bioplastics present higher values compared to common petroleum-based plastics such as polyethylene terephthalate (PET) and polystyrene films with ∼32 and ∼20 g m^–2^ day^–1^ WVTR values, respectively.^50,51^ A clear decreasing trend of WVTR with the hydrolyzed lignin content was observed. The values ranged between ∼9490 g m^–2^ day^–1^ for CLX-0 and ∼7356 g m^–2^ day^–1^ for CLX-50 (i.e., a reduction of ca. 23%). The reduction in WVTR values of lignin-based bioplastics might be ascribed to the role of hydrolyzed lignin as a barrier, increasing the tortuous path for water vapor diffusion, which would result in less permeation of water molecules through the films.^52^ On the other hand, the oxygen transmission rate (OTR) values are reported in Figure 5C. In general, OTR decreased linearly with the hydrolyzed lignin content, from ∼332 mL m^–2^ day^–1^ for CLX-0 to ∼3 mL m^–2^ day^–1^ for CLX-50 (a reduction of ∼99%). The excellent oxygen scavenging behavior of hydrolyzed lignin, as discussed below, can result in a low O_2_ permeability.^52^ A comparison between the OTR values of lignin-based bioplastics and petroleum-based plastics is shown in the inset of Figure 5C.^53^ Lignin-based bioplastics presented competitive values, lower than high-density polyethylene (HDPE) and polypropylene (PP) films, with OTR values of 3000 mL m^–2^ day^–1^ and 2500 mL m^–2^ day^–1^, respectively, and polyvinylchloride (PVC) and comparable or even better than polyethylene terephthalate (PET) films, with OTR values of 150 mL m^–2^ day^–1^ and 75 mL m^–2^ day^–1^, respectively. Water vapor permeability (WVP) and oxygen permeability (OP) were also calculated and are displayed in Figure S4. WVP and OP decreasing trends were also directly related to the increasing amount of hydrolyzed lignin in the samples. CLX-0 showed highest WVP (∼1.7 × 10^–4^ g m^–1^ day^–1^ Pa^–1^) and OP (∼13260 mL μm m^–2^ day^–1^) values, while CLX-50 exhibited very low WVP (∼1.08 × 10^–4^ g m^–1^ day^–1^ Pa^–1^) and OP (∼314 mL μm m^–2^ day^–1^) values. Moreover, the grease resistance test for control samples and for CLX bioplastics was performed using a kit number calculated according to the test described in the Experimental Section. In general, the higher the Kit number, the better are the grease barrier properties of the bioplastics. From the test, it can be reported that control samples C and X have Kit values of 3 and 2 ± 0.5, respectively. However, the increasing hydrolyzed lignin content led to an increase in the grease resistance of the bioplastic samples. In fact, CLX-0, CLX-25, and CLX-50 showed Kit values of 4 ± 0.3, 6 ± 0.5, and 8 ± 0.5, respectively. This behavior could be directly related to the presence of hydrolyzed lignin in the xylan–cellulose matrix. These values are generally acceptable in most packaging applications.

### 3.4 Antioxidant and Antimicrobial Properties

**Figure 6:**
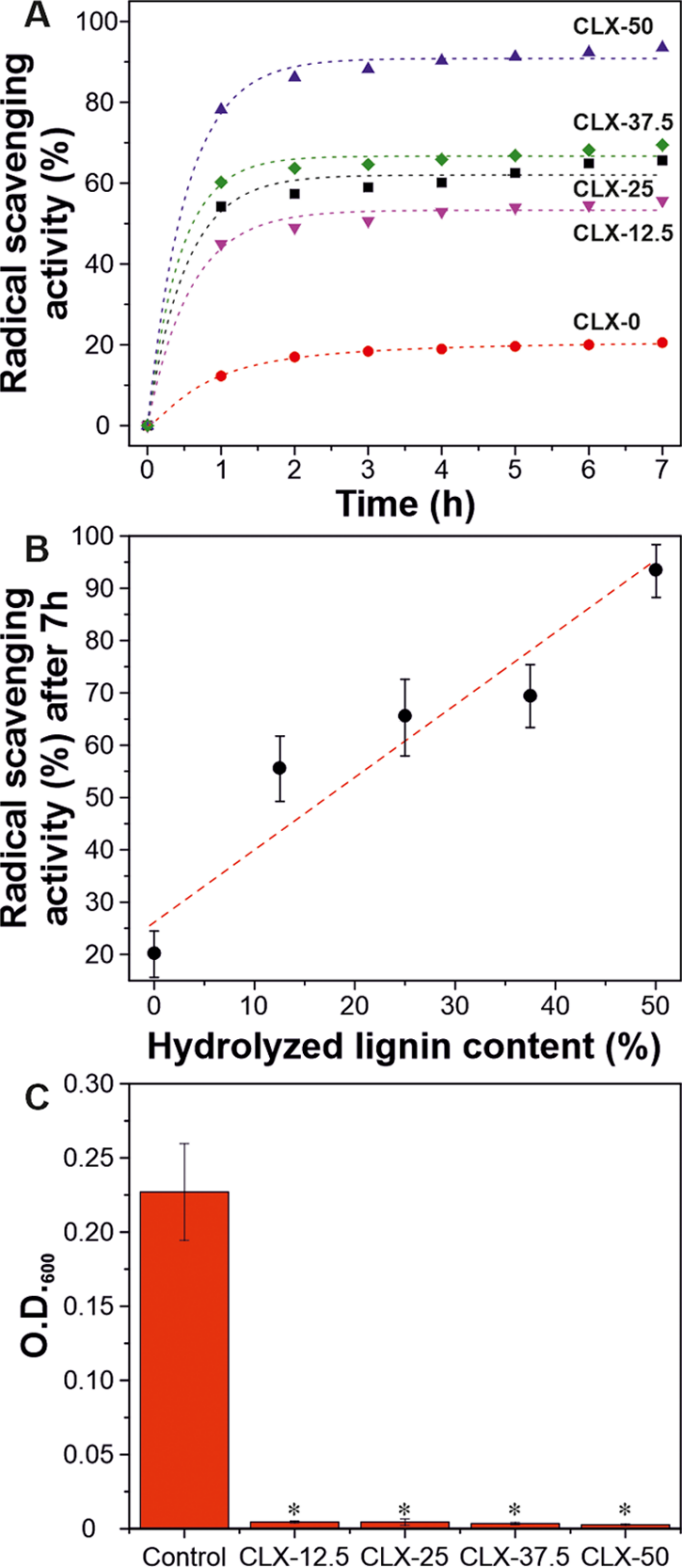
(A) Antioxidant capacity of lignin-based bioplastics with time. (B) Radical scavenging activity values after 7 h as a function of hydrolyzed lignin content. (C) Bacterial growth on lignin-based bioplastics as an indicator of antimicrobial activity (*P < 0.02 vs control).

The antioxidant capacity of the lignin-based bioplastics was tested using the ABTS radical scavenging method. Figure 6A shows the radical scavenging values at different times, while the antioxidant capacity for the controls (C, X, and L) as well as the calibration curve made with Trolox (an antioxidant substance) are displayed in Figure S5. As observed, the antioxidant capacity of the samples was increased with the hydrolyzed lignin content, reaching a plateau after 2 h. According to the calibration curve, the antioxidant capacity of CLX-0 was equivalent to 20 μM Trolox solution, CLX-25 to 68 μM Trolox solution, and CLX-50 to 98 μM Trolox solution. Figure 6B presents the final radical scavenging values at 7 h. Values linearly increased with the hydrolyzed lignin content, from 20% for CLX-0 to 94% for CLX-50. This increase can be associated with the phenolic compounds contained in the lignin fraction able to react with the ABTS radical, avoiding its oxidation.^54^

The antimicrobial activity of lignin-based bioplastics is presented in Figure 6C. *E. coli* is one of the most important pathogens for humans and many animals. It is responsible for a broad spectrum of diseases causing enteritis, urinary tract infection, septicemia, and other clinical infections such as neonatal meningitis.^55^ The infiltration assay to *E. coli* revealed a bacteriostatic behavior for CLX-12.5, CLX-25, CLX-37.5, and CLX-50 compared to the control (bacterial drop). As displayed in Figure 5C, for all lignin-based bioplastics with hydrolyzed lignin, a significant decrease in the O.D._600_ (i.e., absorbance, or optical density, of a sample measured at a wavelength of 600 nm) value can be observed. This result is directly related to the decreased bacteria concentration in the medium after the contact samples are loaded with hydrolyzed lignin, as shown in the inset of Figure 6C. As previously reported, the antimicrobial properties of lignin are directly associated with the nature of phenolic compounds. In fact, these elements damage the cell membranes of microorganism and cause lysis of the bacteria, followed by the release of the cell content.^56^

### 3.5 Biodegradability in Seawater

**Figure 7:**
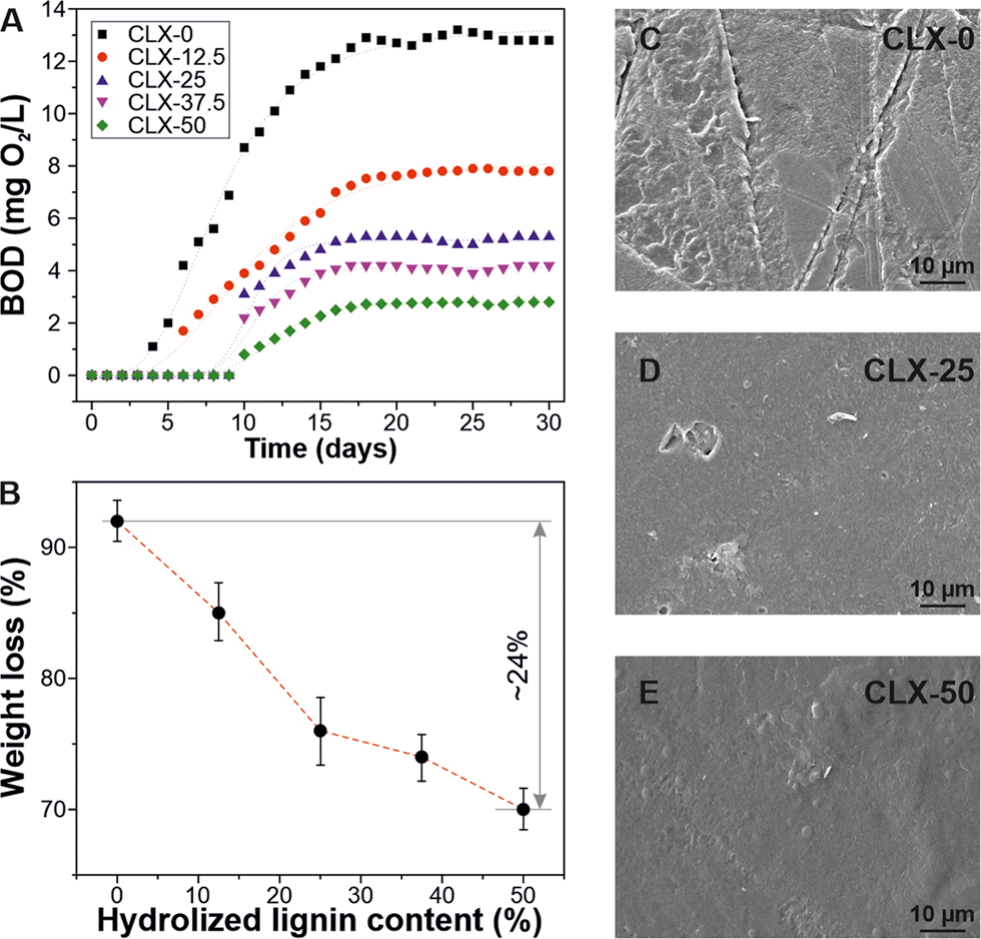
(A) BOD data of lignin-based bioplastics for one month. (B) weight loss after 30 days of BOD tests as a function of hydrolyzed lignin content. (C, D, E) SEM images after BOD tests of CLX-0, CLX-25, and CLX-50, respectively.

To analyze the biodegradability of the lignin-based bioplastics, biological oxygen demand (BOD) was performed. Figure 7A shows the results in a period of 30 days. For the controls (C, X, and L), the BOD curves are shown in Figure S6. As seen, the biodegradability increases with the xylan content. For CLX-0, the biodegradability started after 4 days, achieving a final value of BOD of 12.8 mg O_2_/L. For the sample CLX-12.5, the degradation started after 6 days and the final BOD value was 7.8 mg O_2_/L. For CLX-25, CLX-37.5, and CLX-50, the degradation started after 10 days, while the BOD values were 5.3, 4.2, and 2.8 mg O_2_/L, respectively. The delay observed in the starting time of the biodegradation with the hydrolyzed lignin content can be related to the above described antimicrobial activity of the lignin-containing samples. Thus, microorganisms responsible for biodegradation can die in the initial days until the antimicrobial activity is exhausted. Important differences were also found in the final BOD values of the CLX samples. Thereby, an inverse correlation between the final BOD values and the hydrolyzed lignin content was noticed, phenomena that can be understood by the more structural complexity of lignin fragments in comparison with cellulose or xylan.^57^Figure 7B shows the weight loss after 30 days in seawater as a function of hydrolyzed lignin content. For all cases, the weight loss was higher than 70%. As expected, the weight loss after the BOD experiment decreased with the hydrolyzed lignin content, resulting in a difference of ∼24% from CLX-0 to CLX-50. This tendency can be explained by the different chemistry of the components. Thus, while the polysaccharides biodegrade completely and faster due to specific enzymes (i.e., cellulases and xylanases) secreted by the microbiota in the seawater, lignin (even the hydrolyzed one is used in this study) has a more complex structure, resulting in a recalcitrant material.^58,59^ However, long-term BOD studies have shown that lignin can fully biodegrade in natural waters to form by-products and other decomposable organics that can be metabolized by the microorganisms present in the environment.^57^ Finally, biodegraded samples were morphologically characterized by SEM, Figure 7C–E. The damage was inversely proportional to the hydrolyzed lignin content, most likely because of faster biodegradation of polysaccharides than hydrolyzed lignin by microorganisms.^60^

## 4 Conclusions

In this work, the reassembly process of cellulose, hemicelluloses, and hydrolyzed lignin to fabricate amorphous, free-standing bioplastics with multifunctional properties has been described. The preparation was carried out through the solution of the natural polymer in a mixed system of TFA and TFAA, casting, and complete evaporation of the solvent. The developed bioplastics have been found to cover a broad range of optical and mechanical properties (from relatively ductile to rigid) depending on the final formulation and comparable to common plastic materials. All of the analyzed samples were thermally stable, presenting two main regions of degradation related to hydrolyzed lignin and the polysaccharide phase. The presence of hydrolyzed lignin affected the H-bond network and improved the hydrophobicity as well as water vapor, oxygen, and grease barrier properties. In addition, these bioplastics showed good antioxidant and antimicrobial properties and a high biodegradability in seawater. Nevertheless, further investigations about the oxygen barrier properties with different conditions of RHs of the samples can provide interesting information. To conclude, all features examined make such polymeric materials an attractive alternative to common petroleum-based plastics.

## Supporting Information Available

Photographs of the control materials C, X, and L have been shown. In particular, it could be observed that L does not form a free-standing film (Figure S1); cross-section SEM images of control samples C and X and of CLX bioplastics, respectively (Figure S2); XRD patterns of the control materials C, X, and L with the expected peak position reference for Na_2_SO_4_ (orange, ICSD 98-000-2895) (Figure S3); water vapor permeability (WVP) and oxygen permeability (OP) of all of the bioplastic samples (Figure S4); antioxidant capacity of C, X, and L with the time and the radical scavenging activity values after 7 h for Trolox (Figure S5); biological oxygen demand (BOD) data of the control samples C, X, and L (Figure S6) (PDF)
