# Supplementary material for: Multifunctional Bioplastics Inspired by Wood Composition: Effect of Hydrolyzed Lignin Addition to Xylan–Cellulose Matrices
Source: Biomacromolecules. 2020 Jan 15;21(2):910–20. doi: 10.1021/acs.biomac.9b01569 (PMC7993636; doi:10.1021/acs.biomac.9b01569)
Supplement: Supplementary file 1 — bm9b01569_si_001.pdf [file bm9b01569_si_001.pdf]

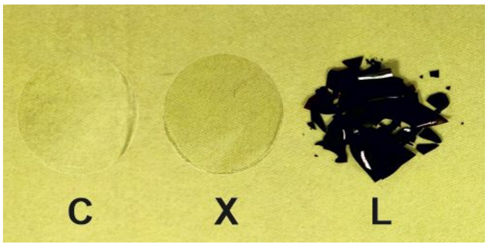

Figure S1. Photographs of the control materials C, X, and L, respectively. As observed, L does not form a free-standing film.

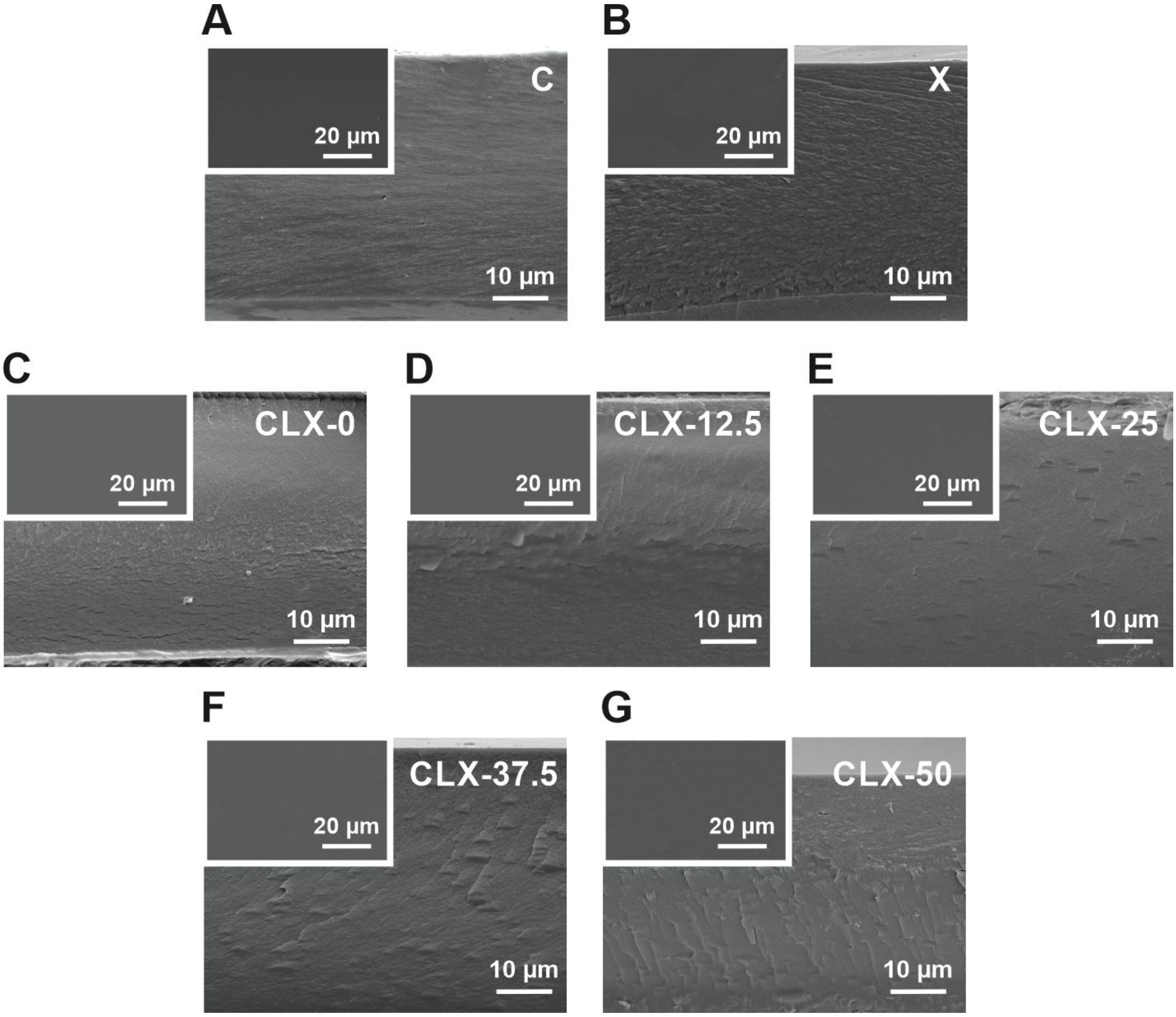

Figure S2. Cross-section SEM images of control samples C and X **(A, B)** and of lignin-based bioplastics **(C, D, E, F, G)**, respectively (scale bar = 10 µm). Insets: corresponding top-views (scale bar = 20 µm).

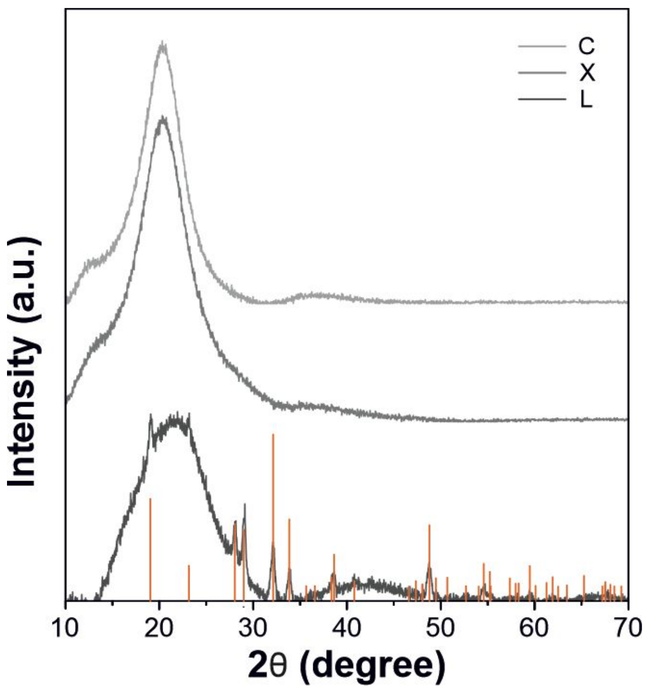

Figure S3. XRD patterns of C, X and L with the expected peak position reference for Na_2SO_4 (orange, ICSD 98–000–2895).

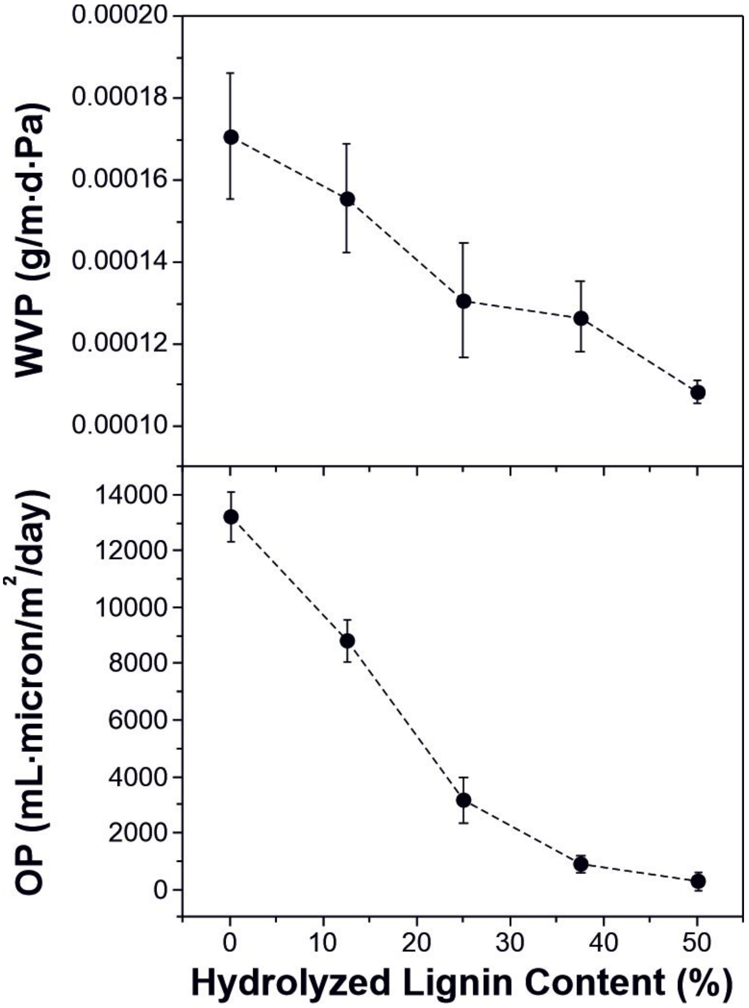

Figure S4. Water Vapor Permeability (WVP) and Oxygen Permeability (OP) of all the bioplastics samples.

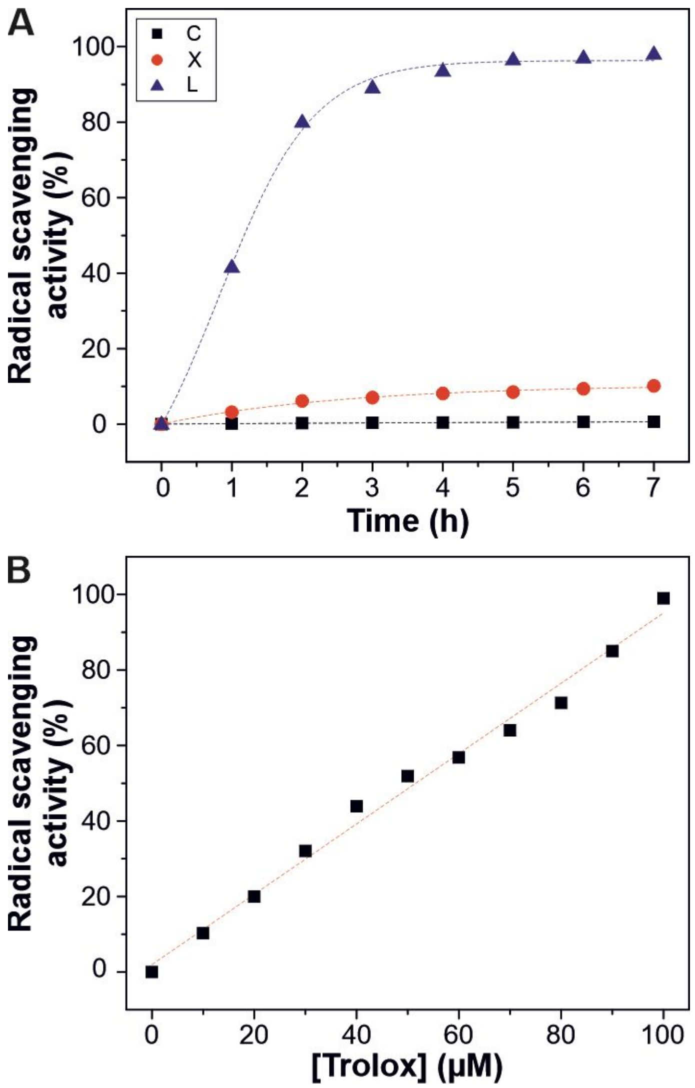

Figure S5. (A) antioxidant capacity of C, X and L with the time. **(B)** radical scavenging activity values after 7 hours for Trolox.

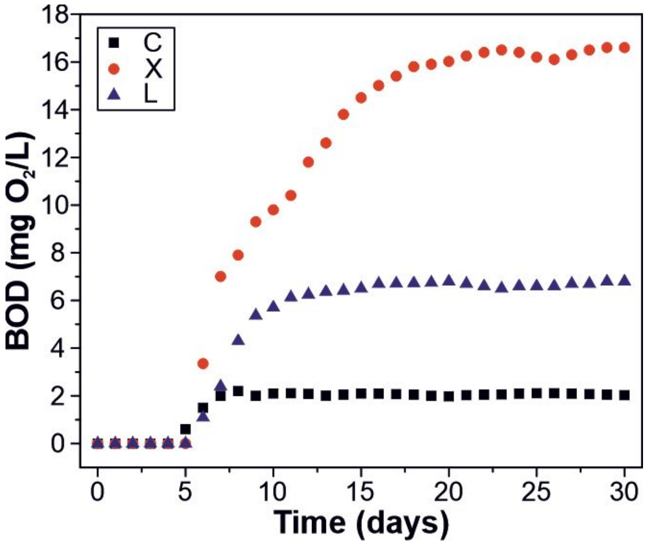

Figure S6. BOD data of C, X, and L.
